# Outcomes of mechanical circulatory support for ventricular tachycardia ablation in severe systolic heart failure

**DOI:** 10.1007/s10840-021-00997-x

**Published:** 2021-05-01

**Authors:** L. Christian Napp, Mir B. Basir

**Affiliations:** 1 Cardiac Arrest Center, Advanced Heart Failure Unit, Department of Cardiology and Angiology, Hannover Medical School, Carl-Neuberg-Str. 1, 30625 Hannover, Germany; 2 Division of Cardiology, Henry Ford Hospital System, Detroit, MI USA

**Keywords:** Ventricular tachycardia, Ablation, Mechanical circulatory support, Impella, Heart failure, Cardiogenic shock

## Commentary

The prevalence, socioeconomic and clinical burden of chronic heart failure with reduced left ventricular (LV) ejection fraction (HFrEF) is exponentially growing [1]. Ventricular tachycardia (VT) frequently occurs in patients with HFrEF, and is not only a phenotype of structural heart disease [2] but may indicate progression of heart failure and risk of decompensation. VT ablation serves as a symptomatic and prognostic therapy in affected patients and has a Class IIa recommendation (level of evidence B) in the European Society of Cardiology guidelines [3]. VT ablation often requires prolonged procedural times, prolonged duration in VT, repetitive endocardial stimulation, and may result in acute hemodynamic decompensation (AHD). The PAINESD score serves as an important predictor of AHD [4] and can help to decide if pre-emptive hemodynamic support is warranted. Although there is encouraging retrospective data on hemodynamic support [5, 6], randomized controlled trials on the utilization of percutaneous ventricular assist devices (pVAD) for VT ablation are lacking, and there is wide variation of use. In this context, we read the study of Chen et al. with great interest [7], yet the conclusions drawn from the results raise several concerns.

The authors analyze a cohort of 69 patients who underwent VT ablation supported with an Impella 2.5 or CP pVAD (Abiomed, Danvers, MA, USA), chosen at the operator’s discretion). The cohort was divided into two groups based upon on duration of support. The early cohort (43 patients) included those who had pVAD removed within 24 h of ablation, and the delayed cohort (26 patients) included those who required pVAD for > 24 h (up to 9 days). Within the delayed cohort, 12 of 26 patients had a change to “other” hemodynamic support devices, while 14 remained on Impella alone. Mortality in the delayed cohort (50.0%) was significantly higher than in the early cohort (2.3%), leading the authors to entitle their manuscript and conclude that “delayed removal is associated with increased mortality.”

A few points to consider:

1. pVAD was employed “per operator discretion;” however, there is no information provided what criteria, if any, were used for employing pVAD support and choosing between devices.
2. The level of shock and hemodynamics prior to the procedure were not reported. Given the heterogeneity of such patients this information is needed. Patients may present with VT storm and cardiogenic shock, or stable HFrEF tolerating medical therapy in a rather elective setting. In fact, the delayed cohort had twice as many patients presenting in VT storm and this difference alone may account for the results of this study.
3. It is not clear if use of pVAD was performed preemptively or as a rescue. This is important because emergent use of pVAD has been shown to be associated with worse outcomes [5, 8, 9].
4. The authors mention that the most prevalent cause of death was progressive shock or refractory HF. It is unclear why 9 patients were switched from Impella to IABP, which provides less support. Similarly of the 13 patients who died, it is unclear why no patient was escalated to ECMO along with the Impella already in place (ECPELLA), which provides robust hemodynamic support.
5. Providing additional information on number and duration of antiarrhythmic medications would be useful, and if prior VT ablation had been performed.
6. It would further be useful to know how patients were sedated, as the use of general anesthesia poses a risk of deterioration and shock for patients with HFrEF, as well as when patients were extubated? General anesthesia for the whole period of support in the delayed cohort would in part explain the worse outcome.
7. Lastly, it would be important to know how patients were weaned from pVAD and if invasive hemodynamics were used. Though the authors mention possible harm with the use of pulmonary artery (PA) catheters, the studies referenced are still controversial and did not include patients with pVAD or patients in cardiogenic shock. In fact, the use of PA catheters has been recently associated with improved outcomes in patients with cardiogenic shock [10, 11]. Unlike percutaneous coronary interventions which predominantly affect the LV, repeated VT will affect both LV and RV function. Therefore, a complete understanding of biventricular function is important in this context and will facilitate early detection of hemodynamic decompensation.

The cohorts differ in several aspects: patients in the delayed cohort had a numerically lower LVEF, more COPD, more chronic kidney disease, more diabetes, higher NYHA class, and a higher PAINESD score. Even if not statistically significant, due to the sample size, the delayed cohort is substantially sicker. These factors contribute to a higher risk and higher heart failure severity of this cohort of patients, which presumably resulted in the longer duration of hemodynamic support. This is also reflected by the four times higher proportion of Impella CP use in the delayed cohort, as treating physicians likely considered these patients to require a higher level of support. Finally, the delayed cohort underwent longer procedures, longer durations on VT, and more ventricular fibrillation induction. Therefore, in the presence of already higher preexisting morbidity, patients underwent more stressful procedures, resulting in the need for longer duration of hemodynamic support. The investigators could consider adjusting for the aforementioned baseline differences or performing a matched pair analysis. Notwithstanding, a prospective study is needed to test outcome effects of prolonged versus limited time on support, in cohorts with comparable pre-existing risk and morbidity.

The mean LVEF in the early and delayed cohorts was 27.1 and 20.6%, respectively; however, many patients were not on optimal medical therapy for heart failure: Only 50 to 60% had an ACEI/ARB, and the use of mineralocorticoid receptor antagonists and sacubitril/valsartan was not reported. Higher intensity medical therapy before the procedure may have resulted in better procedural outcomes. Patients who were not able to tolerate medical therapy, or who had VT storm with poor LV function, might have had refractory end stage heart failure. This would warrant evaluation for advanced heart failure therapies such as durable LVAD or cardiac transplantation, rather than high-risk VT ablation.

Randomized controlled trials on the use of pVAD in interventional or electrophysiological procedures are difficult to conduct. Reporting data from observational studies is therefore essential; however, inherent forms of bias must be considered. Concluding that prolonged support per se is the cause of higher mortality is not well supported by the evidence presented. It is not the time on support, but the underlying severity of heart failure, the existence of comorbidities, and the hemodynamic deterioration of a prolonged and complex procedure which drives morbidity and mortality. Careful patient selection and timing, use of multi-modality heart teams, use of invasive hemodynamics for decision making and monitoring, and individualized mechanical circulatory support strategies are crucial in our efforts to maximize patient outcomes.
